# Direct-to-Consumer Genetic Testing's Red Herring: “Genetic Ancestry” and Personalized Medicine

**DOI:** 10.3389/fmed.2019.00048

**Published:** 2019-03-29

**Authors:** Mwenza Blell, M. A. Hunter

**Affiliations:** ^1^Policy, Ethics and Life Sciences Research Centre, School of Geography, Politics and Sociology, Newcastle University, Newcastle-upon-Tyne, United Kingdom; ^2^Department of Philosophy, Logic, and Scientific Method, Centre for Philosophy of Natural and Social Science (CPNSS), The London School of Economics and Political Science, London, United Kingdom; ^3^Philosophy Department, University of California, Davis, Davis, CA, United States

**Keywords:** genetic testing, ethnicity, race, personalized medicine, ethics

## Abstract

The growth in the direct-to-consumer genetic testing industry poses a number of challenges for healthcare practice, among a number of other areas of concern. Several companies providing this service send their customers reports including information variously referred to as genetic ethnicity, genetic heritage, biogeographic ancestry, and genetic ancestry. In this article, we argue that such information should not be used in healthcare consultations or to assess health risks. Far from representing a move toward personalized medicine, use of this information poses risks both to patients as individuals and to racialized ethnic groups because of the way it misrepresents human genetic diversity.

## Introduction

It may seem surprising now to think that not long ago genetic testing was largely confined to clinical and research contexts. The progressive reduction in the cost of sequencing and genotyping, coupled with the limited demand for clinically-driven testing in the early 2000s led to companies marketing and selling their tests directly to consumers to increase sales (1). Direct-to-consumer (DTC) genetic tests, as the name suggests, most often return results directly to the consumer without the involvement of a clinician and this industry has grown rapidly in the past two decades, despite the concerns of clinical geneticists and genetic counselors (2, 3). In 2016, one study identified 246 different companies who offered some form of an online DNA test, where 74 companies (roughly 30%) offered ancestry services, and 92 (roughly 37%) offered genetic relatedness services (4). According to the MIT Technology Review, “[t]he number of people who have had their DNA analyzed with direct-to-consumer genetic genealogy tests more than doubled during 2017 and now exceeds 12 million, according to industry estimates” (5). Explicitly health-related direct-to-consumer genetic testing emerged initially within an ambiguous regulatory setting in many countries (6). In the US context there were no clear regulatory mechanisms in place to assess the analytical validity, clinical validity, and clinical utility of DTC tests in the early 2000s when sale of genetic tests was beginning to gather speed (6). Between 2015 and 2017 some large scale companies in the industry worked to develop the evidence base to support their medical claims and, as a result, in 2017 the US FDA authorized the first DTC genetic test as an approved medical device, marking the start of a phase some authors have called DTC 2.0 (6). The industry has been criticized for problems with reported invalidity of health-related results (7), lack of data privacy (8), and lack of transparency (9).

There is a definite mismatch between what consumers think that they are getting from these tests and what the companies themselves state they are giving to consumers of their products. This despite the fact a number of companies have issued public disclaimers, stating that “their services are not meant to be used as medical advice or as a diagnostic tool” (10). There is also empirical evidence that concerns about DTC genetic testing are more prevalent for certain minority groups (11). Recent work done by sociologists, anthropologists, and historians illustrates the myriad misunderstandings, miscommunications, and attempts to deal with historical discrimination between medical practitioners and marginalized people that have arisen around the issue of genetic testing (12–14).

One feature of some DTC genetic testing companies' output is referred to variously as biogeographical ancestry, genetic ethnicity, genetic heritage, or genetic ancestry (hereafter genetic ancestry), suggesting the person's DNA has been separated into sections associated with the places or groups from which the DNA originates. The output normally consists of a list of percentages associated with particular continents and/or currently existing countries and/or ethnic/racial groups, depending on the company. In many cases, a primary motivation for consumers' engagement with DTC genetic testing is to receive this information (10). Advertising for a number of these companies strongly suggest they will connect the individual to their own ethnically-inflected history (“Discover your genetic heritage,” “Trace your DNA. Find your roots,” “Explore your genetic roots,” “What's your tribe.”) (15).

Some authors have called for genetic ancestry test results to be used in healthcare, suggesting this would constitute “closing a gap” between health-related genetic testing and genetic ancestry testing (16). Some tests already output ethically-specific disease risk estimates for consumers based on their own proprietary ancestry results, meaning the services are already integrated (17). In this paper, we argue against the integration of DTC genetic ancestry test results into healthcare because of the risks to patients—risks that stem from the invocation of poorly operationalized concepts that misrepresent the accuracy and validity of these tests. We draw upon studies and literature from empirical and theoretical sources that are broadly focused upon DTC genetic ancestry tests, even though some specific contexts (such as the USA and UK) are highlighted., drawing on knowledge from studies of human genetic diversity and social research in the US and UK.

## Genetic Ancestry Test Results in the Context of Human Biology

Genetic ancestry results, with their percentages by region and often slick presentation, certainly provide an appearance of precision to the consumer but this very appearance is “dangerously seductive and equally misleading” (18). The validity of the genetic ancestry results of DTC testing have been questioned and challenged on several grounds. Numerous news articles, blog posts, and YouTube videos attest to inconsistencies in results from different companies' genetic ancestry tests (19–22), even for the same test's results for identical twins (23), and the same test's results at different points in time (24). It has been highlighted in a report to the US Congressional Research Service that the widespread use of proprietary databases by these companies means the scientific claims made in genetic testing results cannot be independently verified (25).

The idea of using genetic data to categorize people into continental groups to further knowledge about differences in disease risk by race or ethnicity is not new (26), nor is the idea that genetic differences underlie differences between white and racialized populations in terms of physiological aspects of diseases that occur in both sets of populations (27–29). However, a large and long-standing body of research refutes the supposition that epidemiologically-evidenced ethnic differences in human disease risk, including diseases with a demonstrated genetic basis and limited geographic distribution, are evidence in support of the notion of biological race (30, 31). Indeed, repeated studies of human population genetics for well over 40 years have concluded that the genetic data do not support the notion of biological races in humans (29, 32–34) and some geneticists have called for an end to the use of racial and ethnic categories in clinical contexts on this basis (29).

Despite this, attempts to reinscribe the notion of biological race in humans continue, particularly in biomedical and biotechnology arenas (31, 35, 36). Legal scholar Kahn notes an increasing trend in conflation of race and genetics in gene-related patent applications, citing “the strategic use of race as a genetic category to obtain patent protection and drug approval” (37). Some have specifically argued that explicit racial profiling is a positive step toward the much-promoted ideal of personalized medicine based on the logic that racial groups differ genetically (38). Indeed a tendency has been noted in the conceptualization and implementation of personalized medicine, the tendency for this to become racialized medicine, with racial categories used as proxies for specific functional genetic information (36, 39). Because of what we know about the way human genetic diversity is apportioned, any scientific inference made which assumes humans come in “neat racial packages” is not trustworthy and, as a consequence, the only valid way to understand what is in a person's DNA is to study that individual's DNA (32). Assumptions about disease that are made because of a racial classification can result in important negative consequences for individual patients and inaccurate genetic inferences for populations (31).

In order to make the meaning of the above statements clearer, we expand on them here. Barbujani and Pigliucci have pointed out that any two groups of human beings, divided on any basis, however arbitrary (e.g., plumbers vs. dentists), will differ to some extent in average measures of any particular biological trait one chooses, including genetic traits (e.g., ability to digest milk), and this does not mean these groups represent important taxonomic categories (or that ability to digest milk influences choice of plumbing over dentistry as profession) (32). As Lee et al. write in Statements 2 and 7 of their ethical principles:

“Statement 2: We recognize that individuals of two different geographically defined human populations are more likely to differ at any given site in the genome than are two individuals of the same geographically defined population Research in human genetics has highlighted that there is more genetic variation within than between human groups, where those groups are defined in terms of linguistic, geographic, and cultural boundaries … Statement 7: We discourage the use of race as a proxy for biological similarity and support efforts to minimize the use of the categories of race and ethnicity in clinical medicine, maintaining focus on the individual rather than the group Although a broad range of associations between genetic markers and human traits—including diseases—is emerging, any accompanying correspondence with race or ethnicity is statistical.” (29).

Even Feldman et al. who suggest that identifying “all contributions to a patient's ancestry can be useful in diagnosing and treating diseases with genetic influences,” a point with which we disagree on the basis that all ancestral contributions cannot, in fact, be identified (see below), note that “attempts to classify people into broad genetic groups based on the frequency of specific genes for, say, drug-metabolizing enzymes, are also likely to be poor predictors of medical outcome (40). As with racial groupings, the overall variation in the frequencies of such genes between groups is likely to be less than that within each group” (40).

Since there is no deterministic connection between being part of a group of people, whether referred to as a population, ethnic group, or race, and a particular genotype, genetic ancestry-based approaches to individual care lack an appropriate theoretical basis (35) and are thus a risk to individual patients. Although consumers may approach doctors with the results of their genetic ancestry tests, requesting that these be taken into account, doctors should exercise great caution (41), consider the evidence such as that outlined in this paper, and hopefully conclude that an ethical and truly personalized medical practice should mean abandoning invalid ethnicity- or race-based assumptions about individuals' relevant genetic characteristics.

### Genetic Ancestry Test Results: Debunking “Ancestry”

The notion of ancestry itself deserves some attention. The term has inherent temporal ambiguities which others have highlighted (42). The ancestry tests purport to give information about ancestry from a time before the grandparental generation but certainly after the distant time of the first transcontinental migrant hominin groups since all ancestry results would be African from that time standpoint (34). Yet the potential of these tests has hinged upon the notion that there is a coherent understanding of the term “ancestry.” The term is deployed throughout the literature, however, upon further examination we find that there are important conceptual problems with its use.

One clear example of this comes from the landmark Rosenberg et al. paper on the structure of human populations (42). In their paper, the authors utilize a number of different conceptions of the term “ancestry” without providing any sort of robust operationalizable definition of the term. Included in the article are “self-reported ancestry,” “genetic ancestry,” “Mongol ancestry,” “self-reported population ancestry,” and “genetically inferred ancestry” (42). The use of these terms without any sort of clarification is problematic. How exactly are medical practitioners and biomedical researchers supposed to understand the classificatory permutations of the concept *ancestry*?

One of the only attempts to clarify what the term “ancestry” means is found in Via et al. (43). In their article, however, their attempts to clarify the term fail to make any substantial progress. To note, the authors state that it is important to find a different term to use in place of “race” or “ethnicity” for biomedical research and the authors propose the use of genetic ancestry as an alternative, of which DTC ancestry kits are ostensibly a developing part. Via et al. (43) proceed to state that their definition can be defined on several levels: *biogeographical* (i.e., African vs. Asian); *geographical* (i.e., south-east Asian vs. northern European); *geopolitical* (i.e., Cambodian vs. Swedish); and *cultural* (i.e., Jewish vs. Berber) (43). Additionally, they state that determinations of ancestry can *also* be varied: they can be self-identified; identified by an observer; or estimated from genetic data. Lastly, in an attempt to provide a final clarification, the authors state that ancestry can be defined by one or multiple sources. The definition given by Via et al. gives rise to a number of downstream complications that have specific ramifications upon both clinical practice and biomedical research.

One set of complications is methodological: if Via et al. are correct that determinations of ancestry can be varied, which will win out when one or more of these descriptions of ancestry are in conflict? If ancestry can be determined by an observer, how exactly does a clinician or biomedical researcher become one without importing the flaws that the authors find inherent in the use of either “race” or “ethnicity”? The second set of complications is conceptual: What are the agreed-upon classification schemes for biogeographical, geographical, geopolitical, and cultural ancestry? How are they determined given that from an empirical standpoint there is a large overlap between the four aforementioned definitional levels? Is there a hierarchical ordering of the definitional levels, and if so, what ensures that we can make inferences of import for biomedical and clinical outcomes between those levels and individuals? How is the concept *cultural* ancestry, as used in biomedical research and clinical contexts, supposed to work given that Via et al. note that culture is categorically different than an individual's *genetic* background? Given the varied ways in which the term “ancestry” has been used by researchers and clinicians themselves, and that there is no coherent, operationalizable definition upon which clinical and biomedical research has agreed, the use of “ancestry” for anything of empirical import based on DTC products is in serious doubt.

Indeed the term ancestry is inherently misleading, no actual ancestral populations have been (or could be) sampled. The methodological assumptions about past population “purity” and “admixture” are just that—non-empirical assumptions that are at odds with the reality that ancestral populations (which DTC companies purport to report on) cannot be sampled with accuracy. The direct-to-consumer tests at best provide limited support for linking a consumer's DNA profile to places in which individuals whose DNA samples have been analyzed and included in the company's datasets lived at the time of sampling. The reason why the information generated by DTC companies provides limited support is due to the underlying methodology of ancestry studies based on populations. These programs are largely modeled after the program STRUCTURE used in Rosenberg et al. (42, 44), even though there is controversy as to whether STRUCTURE is a reliable program for identifying genetic clusters (45). In short, a baseline data set is generated by using samples from contemporary individuals that have been carefully selected on the basis that they themselves are members of a “pure” or “non-admixed” population or a “pure” or “non-admixed” individual. For individuals this would require, for example, that the only people allowed to be part of a “non-admixed” population are those whose four grandparents are also part of that same population.

Of course, present-day patterns of residence are rarely identical to what existed in the past due to historical events—both social and natural—that have influenced patterns of migration. Humans have migrated to such an extent that many individuals around the world have relatively recent ancestors from far-flung regions (46). As a result, databases of present-day samples, regardless of reporting about grandparents, can provide false leads (47). In addition, false assumptions about the distinctiveness of populations in the past appear to influence genetic ancestry analyses (46). As Duster notes, the notion that a population group is “pure” or “non-admixed” “is a statistical artifact that begins not with the DNA, but with the researcher's adopting of the folk categories of race and ethnicity”(48). Given what is common knowledge about evolutionary biology, there is no such thing as a “pure” population in the mathematical sense. It is common knowledge for demographers, epidemiologists, and other social scientists that the terms used to describe racial/ethnic groups have changed across time (49, 50), especially in particular contexts like the UK and the USA, where the changes in official racial/ethnic categories over time have been particularly stark. If epidemiologists and other health practitioners are focused on dealing with the patterns and causes of disease (e.g., Type 2 diabetes) across contexts, there will be a mis-match between operationalized groups in different geo-political regions. The people who count as “white” in one region may not neatly count as “white” in another region or at another time period, thus hampering the ability to detect valid and reliable epidemiological relationships, much less associations of significance to direct care (51).

The analytic techniques used in genetic ancestry tests clearly focus on the small differences between current populations rather than similarities between populations. Studies looking to estimate genetic clustering have tended to choose samples from places both more geographically and socially distinct from one another. When samples have been collected using more even geographical sampling, clustering has been “far less evident” (46). This is because human genetic variation is typically clinal. As Duster points out “when researchers claim to be able to assign people to groups based on marker frequency at a certain number of loci, they have chosen loci that show differences between the groups they are trying to distinguish.” Given that populations can be demarcated by a number of criteria [see Kaplan and Pigliucci 2003 (52)], the focus upon difference is not only misleading about human genetic variation (33) but helps to reproduce and reinscribe the notion of biological race. A further complication is that biases in sampling and analyses mean people living in and coming from the Middle East, South Asia, Southeast Asia, East Africa, and the Mediterranean region of Europe are most likely to receive ancestry results which are less valid, based on their known ancestry or any historical or archaeological evidence (34, 41).

As distinct from these individual- consumer-focused commercial practices, when inferences are made about ancestry in academic research these are the level of the group and created with measures reflecting their fundamentally probabilistic nature (34). Indeed, due to the limitations of DNA itself, regardless of technique, one would not be able to demonstrate genetic links to many of one's actual direct ancestors even if they had been sampled so non-probabilistic measures are impossible (34).

### Genetic Ancestry Tests, Racialized Groups, and Justice

It is worth highlighting the important implications of use of genetic ancestry in terms of the ethical principle of justice in medical practice. In particular, there are considerations specific to racialized ethnic groups. While the concept of “justice” can be construed and understood in quite a narrow way—we wish to emphasize that there are historical and group-level concerns for people who have been marginalized. With respect to medical interventions, the examples of medical injustices that have been perpetrated at the group level include the Tuskegee Syphilis Study (53) and the Puerto Rican Birth Control study (54).

Reinscription of the notion of biological race in medical consultation, even inadvertently, validates the idea that race and ethnicity are natural classifications and runs the risk of encouraging racial/ethnic stereotypes and oversimplifications of the complex origins of most disease, leading to both a naïve genetic essentialism and a misunderstanding of human genetic diversity in society (29, 46). In addition, the effective disregarding of social production of health models of understanding the ethnic patterning of ill-health, which have been developed on the basis of epidemiological research over the past decades, in favor of an assumption of a genetic basis, leaves ethnic groups' own supposedly shared faulty genes as the supposed cause of their ill health. As legal scholar Roberts points out “The biological explanation for racial disparities provides a ready logic for the staggering disenfranchisement of people of color through mass incarceration and other punitive policies, as well as the perfect complement to color-blind policies implementing the claim that racism has ceased to be the cause of their predicament” (36).

For their part, according to some reports, white supremacists groups and individuals have seized genetic ancestry results and publicly used these to argue for scientific racism when they interpret the evidence as in favor of their ideology (55–57), while working hard to delegitimizing any results which threatened their self-identities (56). In the context of the rise of the far right globally and recent reports of scientific racism and eugenics in some of the world's top ranked universities (58, 59), the risk that doctors and medical systems may perpetuate injustice cannot be ignored. Indeed, it has been found in previous research that doctors often lack sufficient knowledge to tackle misinformation associated with DTC genetic tests in general (60, 61). Doctors may be like patients in failing to realize that tests are probabilistic (41) and medical training may not have prepared them with a detailed understanding of human genetic variation (61).

## Conclusion

Given the issues that we have highlighted above regarding DTC products for assessing ancestry for biomedical and other purposes, we wish to reiterate the following important points. The first is that “ancestry estimates,” as currently described in the literature, act as a reinscription of biological race. This is because of the inherent confusion that abounds regarding the concept “ancestry”—both for clinicians, biomedical researchers, epidemiologists, population biologists, and especially the general public. Since there are no principled, operationalizable definitions of “ancestry” (nor “population” for that matter), clinicians, biomedical researchers, and the general public frequently project naive racial categories onto their findings.

Secondly, it remains important to reiterate that medical practitioners—the intermediaries between consumers and potential medical interventions—have incredible obstacles that prevent them from giving their patients a better assessment and understanding of the “ancestry data” that are gleaned from DTC products. In a recent survey by Baer et al. (62), the authors noted that health researchers were “confused about concepts of race and ethnicity and their link to genetic differences between populations; many treated these concepts as interchangeable and genetically based…[and] the younger health researchers tended to put a stronger emphasis on the genetic aspects of race than did the older health researchers” (62). Couple this with the confusion over the concept of “ancestry” that is exemplified by Via et al. and the statement by prominent population biologist Neil Risch [2005] that he “does not even know what race means,” and we can see there is an incredibly dense conceptual field for medical practitioners to attempt to sort out by themselves (63). Given the other constraints on time and energy that are in play, we are loathe to believe that this conceptual clarity will be absolved solely by the community of practitioners that are swamped with this morass.

Our final point is the following: the use of DTC products for assessing the “ancestry” of individuals has a number of ethical issues that are in urgent need of resolution. It would have been better to have thought through and dealt with these issues before the deployment of DTC products rather than after: the ethical issues include potentially providing misleading or inaccurate information that affects the healthcare decisions by practitioners; confusion about differences between ancestry and genealogy for consumers, clinicians, and biomedical researchers; and the reintroduction of racialized concepts under the guise of “genetic ancestry” which has the potential for harming both individuals and marginalized populations. We recommend that the professional bodies of clinical practitioners (e.g., American Medical Association, the Royal College of Physicians) and agencies evaluating medical devices (e.g., FDA, European Medicines Agency) examine the evidence we discuss here and consider issuing specific guidance or warnings about use of genetic ancestry test results in healthcare since these are not suitable for diagnostic purposes and pose risks to individuals and society as we outline above.

We have noted that DTC products are of interest to consumers because of how they are marketed and what they promise to potential users: Ancestry.com promises to “Reveal the places you're from”; in reference to their products, 23andme.com offer “Two easy ways to discover you.” The promissory allure of helping people to “find themselves” by giving a swab of their bodily tissue masks the fact that there are conceptual inconsistencies, methodological shortcomings, and ethical issues that influence whether the general public thinks about personalized medicine as racialized medicine.

## Author Contributions

Both authors drafted the manuscript and readied it for journal submission. MB conceived the paper and invited MH to contribute.

### Conflict of Interest Statement

The authors declare that the research was conducted in the absence of any commercial or financial relationships that could be construed as a potential conflict of interest.
